# The benefits of Q + PPGIS for coupled human-natural systems research: A systematic review

**DOI:** 10.1007/s13280-022-01709-z

**Published:** 2022-03-07

**Authors:** Malcolm S. Johnson, Vanessa M. Adams, Jason Byrne, Rebecca M. B. Harris

**Affiliations:** 1 School of Geography, Planning, and Spatial Sciences, University of Tasmania, Hobart, TAS 7000 Australia; 2 Centre for Marine Socioecology, Institute for Marine and Antarctic Studies, College of Sciences and Engineering, University of Tasmania, Hobart, TAS Australia; 3 Geography and Environmental Studies, University of Tasmania, Private Bag 78, Hobart, TAS 7001 Australia

**Keywords:** Environmental management, Mixed-method, Planning, PPGIS, Q-method, Socio-ecological system

## Abstract

Managing complex problems in socio-ecological systems (SES) requires innovative approaches, which account for multiple scales, large datasets, and diverse lived experiences. By combining two commonly utilized mixed-methods, public participation GIS (PPGIS) and Q-method (Q), Q + PPGIS has the potential to reveal competing agendas and reduce conflict, but its benefits and weaknesses are comparatively understudied. Using a systematic review, we evaluated how different studies have employed and implemented the Q + PPGIS method. We found 16 studies, comprising 30 publications, with considerable variation in their geographic foci, research disciplines, and addressed SES challenges. These studies exhibit a lack of cohesion between methodological design and implementation and the absence of a consistent application of the method. Nonetheless, Q + PPGIS offers a tool that can guide policy, better inform stakeholders, and reduce conflict based on misconceptions. Resolving the shortcomings identified here will broaden Q + PPGIS utility in geographically situating and representing multiple realities within complex socio-ecological systems challenges.

**Supplementary Information:**

The online version contains supplementary material available at 10.1007/s13280-022-01709-z.

## Introduction

Environmental managers, land use planners, and conservation scientists currently face a complex array of socio-ecological problems, including deforestation, biodiversity loss, wildfire and coastal erosion, and flooding, many of which are exacerbated by climate change. These issues are typically defined by complexity and require the application of interdisciplinary methods and skillsets—to both understand the problem and to generate effective policy responses (Redman et al. 2004; Ostrom 2009). Addressing complex socio-ecological problems like these increasingly requires deeper and more nuanced understanding of geospatial, ecological, and socio-cultural knowledge (Hügel and Davies 2020). Solutions to such challenges are often informed by spatially-oriented values, expressed through multiple worldviews.

Because socio-ecological system (SES) problems typically exhibit complex patterns and processes, they are often described as messy and wicked (McGinnis and Ostrom 2014). For this reason, methodologies used to study them need to be able to handle interconnected uncertainties (Liu et al. 2007; Zellner and Campbell 2015). Moreover, such problems are often location-specific but manifest across large spatial and temporal scales, necessitating the use of appropriate geospatial technologies (Singh et al. 2013; Holzer et al. 2018). Potential solutions also include a diversity of worldviews that sometimes, but not always, conflict, so researchers need to find innovative ways to integrate, respect, and respond to diverse perspectives, without diminishing the complexity of the SES challenge or the interrelated human values (Tàbara and Chabay 2013; Pickett 2013). Scholars and practitioners are increasingly calling for more sophisticated, transdisciplinary, mixed-methods approaches that provide the capability to integrate qualitative and quantitative data sets, and are responsive to, rather than blind to, personal subjectivity (Eden et al. 2005; Zabala et al. 2018; Sneegas et al. 2021). To this end, many researchers employ participatory mixed-methods approaches capable of integrating sociocultural and biophysical variables (Alessa et al. 2008), synthesising large geospatial datasets for practical application (Magliocca et al. 2018), and involving multiple stakeholders in decision-making processes (Villamor et al. 2014).

Geographic information system (GIS)-based methods are particularly useful for understanding the spatial-orientation of SES challenges (O’Sullivan 2006). The use of public participation methods, which aim to include community members in the science or policy making process, have been shown to be beneficial because they can reveal common interests (Rowe and Frewer 2000; Webler et al. 2001). The use of qualitative methods, such as Q-method, interviews, focus groups, or surveys, can also lead to the identification of differing and diverse worldviews (Bernstein 2020). These three approaches have been integrated in various ways including GIS-based qualitative analysis, combined ethnographic techniques with quantitative spatial analysis, and qualitative sociological research supported through statistical analyses (Nightingale 2003; Elwood 2010; Ramlo 2016). In the past, Q-method,public participation, and GIS have all been identified as independent participatory data collection methods within natural resource management and social-ecological systems (Colvin et al. 2016; Vos et al. 2021).

Another method, which we will call Q + PPGIS, offers the promise of contextualising stakeholder values and perceptions within and across scales, while incorporating local knowledge and ecological assessment into spatial decision-making and participatory planning. Previously, the method was called “Q + GIS” by Forrester et al. (2015), however, since there is no direct relationship between Q + GIS and the open-source GIS program QGIS, where the “Q” is based on its original name “Quantum GIS” and not Q-method, we found this name confusing, particularly when searching the literature. More recently, Lee (2022) sought to differentiate traditional Q, which they call “general text/statement Q”, from “place/spatial Q”, which uses Q-method for spatial planning, and “combined Q”, where both statement Q and place Q are used for planning research. However, this distinction limits the number of papers that can be considered combined Q, as the two distinct Q approaches need to be utilized rather than any form of Q-method plus GIS in a public participatory planning setting.

**Fig. 1:**
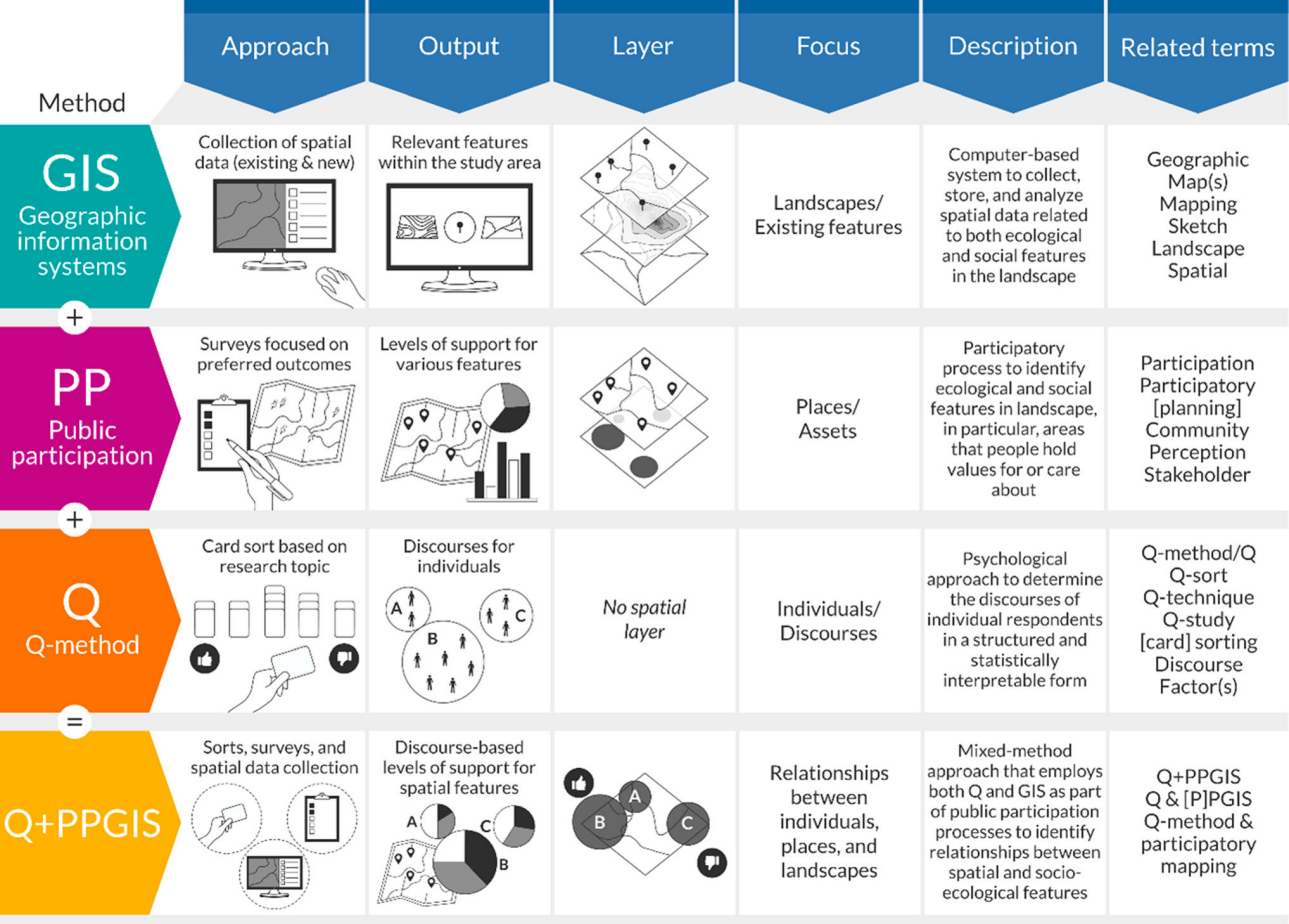
The Q + PPGIS nexus of geographic information systems (GIS), public participation (PP), and Q-method (Q) with their associated approaches, outputs, spatial layers, area of focus, description, and related search terms

The Q + PPGIS method, which combines public participation, GIS, and Q-method (Fig. 1), is potentially well-suited for the robust statistical analysis associated with quantitative methods, the geospatial examination of GIS-based assessments, and the concentration on individual subjectivities sought by qualitative techniques (Robbins and Krueger 2000; Forrester et al. 2015; Druschke et al. 2019). All components of Q + PPGIS have well-established methodological approaches and abundant applications within coupled human-natural systems research, despite shortcomings commonly addressed within the literature, such as the inability of PPGIS to recognize multiple realities or the lack of geospatial positioning of Q-method (Craig et al. 2002; McKeown and Thomas 2013). When combined, this novel approach Q + PPGIS offers a pathway for mixed-method research to gain deeper understandings of the complexities and connections between individuals, communities, and places within socio-ecological systems. However, Q + PPGIS is less well known by researchers, begging important questions—how often has Q + PPGIS been used in SES studies, for what purpose and how successful have its applications been? This paper addresses those questions.

## Background

Integrative approaches which mix qualitative and quantitative methods require diverse skills and expertise that can limit the accessibility and feasibility of mixed-methods participatory research approaches for researchers and practitioners (Hügel and Davies 2020). Researchers often have to choose between well-established methodologies that are efficient at determining patterns between variables or that concentrate on individuals and their experiences, rarely finding space for both within the constraints of project frameworks (Robbins and Krueger 2000). A commonly utilised mixed-method in socio-ecological systems research is participatory GIS-based qualitative analysis. This method, which enables local knowledge held by research participants to be given equal consideration as expert geospatial data, is generally labelled public participation GIS (PPGIS). Building on the first computerised GIS developed by Tomlinson in 1963, PPGIS seeks to integrate qualitative and quantitative information (Craig et al. 2002).

PPGIS is defined as the combination of technology-based spatial analysis from GIS and public participation approaches central to collaborative-, community-, and participatory-based processes that provide multiple means to interpret complex data. PPGIS methods usually follow a three-phase process (Craig et al. 2002): (1) identifying existing data (problem identification, gathering or collecting spatial data, generating a geospatial database); (2) data collection and analysis (developing a participatory mapping approach, interviewing/surveying participants, conducting the mapping exercise); and (3) communication of results (integrating qualitative and quantitative data, developing appropriate data outputs). In some instances, the process has been adapted to be both more iterative and reflexive to better bridge the divide between the qualitative and quantitative (Kyttä et al. 2013; Babelon et al. 2017).

PPGIS has been used in a range of contexts such as protected area management, national forest planning, mapping ecosystems services, and community urban development studies (Brown and Kyttä 2014). These contexts are centred around the benefit of public involvement and participation activities to improve equity of access and stakeholder engagement, thereby reducing conflict, increasing representation, and increasing community buy-in for controversial planning proposals (Schlossberg and Shuford 2005). For example, the method has been used to highlight the differences in perceptions and knowledge of mangroves and ecotourism to better inform forest conservation strategies (Satyanarayana et al. 2012). PPGIS has also been applied to assessments of cultural ecosystem services across local and national scales, to inform policy around climate change mitigation planning (Jaligot et al. 2019).

However, PPGIS is not without its issues. Public participation GIS often relies on highly technical tools that can limit community engagement, due to unequal access to resources (e.g., computer access and digital literacy) (Robinson et al. 2017). Paper-based “offline” mapping methods can encounter similar barriers, based on a community’s awareness of information sources and their ability to apply the GIS outputs (e.g., Indigenous local knowledge in “resource-poor” communities) (Elwood and Leitner 1998; Benyei et al. 2020). Even the best-designed PPGIS studies can struggle with the spatial accuracy and completeness of the data (Brown et al. 2015). For example, if researchers choose to engage stakeholders in group settings, rather than individually, the aggregation of data can misrepresent perspectives, lead to blind spots in data outputs, or result in a combination of groupthink or dominance (Mukherjee et al. 2018). The latter is acutely illustrated in group-based PPGIS settings with consensus-building as a key methodological aim, wherein the interests of a particular individual or group can begin to outweigh underrepresented voices (Schlossberg and Shuford 2005). These issues have driven researchers to augment the capabilities and limitations of PPGIS with qualitative methods—such as in-depth interviews, ethnographic observations, or Q-method—to identify the “patterns in stakeholder perspectives” (Nijnik and Miller 2017).

One approach that seeks to understand the viewpoints of stakeholders around contentious concepts while alleviating potential conflict is the Q-method(hereafter Q) (Albizua and Zografos 2014). Q was developed in the 1930s as a psychological tool to study subjectivity through empirical means (Stephenson 1935). Q has since crossed disciplinary divides and been utilized for exploring policy decisions for an increasingly wide range of socio-ecological issues, including ecosystem service management, disaster risk planning, and coastal/marine governance. Despite the evolution of the method over the last ninety years, Q continues to adhere to a four-phase process (McKeown and Thomas 2013): (1) research design, which includes concourse setting (collecting possible statements people might make), Q-set development (a set of statements that will be used when sorting), and selection of P-set (the participants; (2) data collection, involving conducting Q-sort (placing Q-set within a grid), simultaneous or post-sort interviews, and possible participant surveying; (3) data analysis, through a combination of principal component analysis, maximum likelihood method, or rotated factor analysis to establish the minimum number of factors that account for the maximum variance; and (4) interpretation of the data by establishing the discourses and providing factor narratives.

The primary benefits of Q are that the method allows the identification of patterns in subjective data, such as points of view or discourses, between and across individuals, rather than *of* individuals or groups, thus enabling a focus on central themes and illuminating blind spots (Robbins and Krueger 2000). The analysis can reduce uncertainty around policy decision support (or rejection), linking perspectives with particular discourse groups (Eden et al. 2005). For example, Hawthorne et al. (2008) study on opinions of converting rails into trails led to the identification of community members who opposed the transition, the specific perceptions informing their opposition (e.g., fears about safety), and a recognition of beliefs that were misguided or mistaken. Similarly, Q has identified opposing viewpoints around rodent outbreaks, reducing social tensions arising from management approaches, and allowing more effective decision-making (Lauret et al. 2020). The method has also seen benefits in exploring social values around ecotourism to ensure policy makers implement projects that are supported by the community (Rodríguez-Piñeros and Mayett-Moreno 2015). Q takes the analysis of subjective perspectives and communicates it in a way that can guide policy, better inform stakeholders, and reduce conflict based on misconceptions.

Critics of Q often cite three major concerns with the method: (i) the method’s debatable claim to objectivity, (ii) the considerable degree of researcher bias occurring within each of the four phases, and (iii) a lack of spatial orientation in reporting (Robbins and Krueger 2000). Historically, the method has relied on the assumption that responses from individual participants represent their operant subjectivity (Druschke et al. 2019). While Stephenson (1935) and others boasted this claim to objectivity with Q, in more recent studies this claim has been abandoned in favour of viewing the individual responses as solely points of identification (Druschke et al. 2019). The second critique of Q is that there is a high degree of researcher bias in all phases of the method that is hard to mitigate throughout the method. While some of this bias can be reduced by mixing methodologies, many Q researchers explicitly acknowledge the innate bias of the method and let the value of the results speak for themselves (Eden et al. 2005; Dziopa and Ahern 2011; Ramlo 2016). The third criticism is that Q is often deficient in any spatial orientation or analysis, which when applied to spatially bounded contentious challenges, such as river catchment management or forests ecosystem management, can limit the potential policy recommendations without further research (Cheng and Mattor 2006; Meo 2007). Therefore, commentators suggest that Q-method should be combined with other more common socio-ecological systems tools without diminishing its role to purely a supplemental approach (Eden et al. 2005).

An obvious solution to redress some of the above issues would be to combine Q and PPGIS (Q + PPGIS). Forrester et al. (2015) suggest this could make clear the “connection between what people say and the underlying feelings and values that guide action and behaviour.” The benefit is that Q can investigate shared perspectives while PPGIS can orient them within landscapes (McKeown and Thomas 2013). Yet there is a notable knowledge gap about how socio-ecological research has combined these two methods, and about the potential benefits and limitations of doing so, presenting a challenge for researchers and practitioners.

This paper seeks to remedy that knowledge gap by identifying how, where, and when the mixed-method Q + PPGIS has been applied. We define Q + PPGIS as a *mixed-method approach that employs both Q and GIS as part of public participation processes* (Fig. 1). Utilising a systematic quantitative literature review (SQLR) (Pickering and Byrne 2014; Pickering et al. 2015), we aim to evaluate how different studies have employed and implemented the Q + PPGIS method. The systematic review interrogates several interrelated questions: (1) to what extent have studies utilised Q + PPGIS, and in which fields are they most common?; (2) what are the similarities and differences between various Q + PPGIS approaches?; (3) what are the main benefits and challenges with implementing Q + PPGIS?; and (4) how can Q + PPGIS be applied more thoroughly both within and beyond the current literature?. Following a brief overview of our systematic review method, we identify our key findings and then discuss the importance of these results for both research and practice.

## Methods

To assess the full extent of studies utilising Q + PPGIS methods, a systematic quantitative literature review was undertaken, following the approach detailed in Pickering and Byrne (2014), Pickering et al. (2015), and Pickering et al. (2021). An SQLR entails systematically searching for existing literature with the use of key search terms to classify papers for inclusion, centred on clearly articulated reproducible criteria. The 15-stage process is particularly relevant for studying the extent of Q + PPGIS literature because Q + PPGIS is a nascent method, and the overall utility and efficacy of Q + PPGIS application is not well understood. An SQLR offers a comprehensive, replicable approach to identify knowledge gaps and generate data-supported findings about the application of the Q + PPGIS method in SES studies, including strengths, weaknesses, limitations and prospects.

### Search strategy

**Fig. 2:**
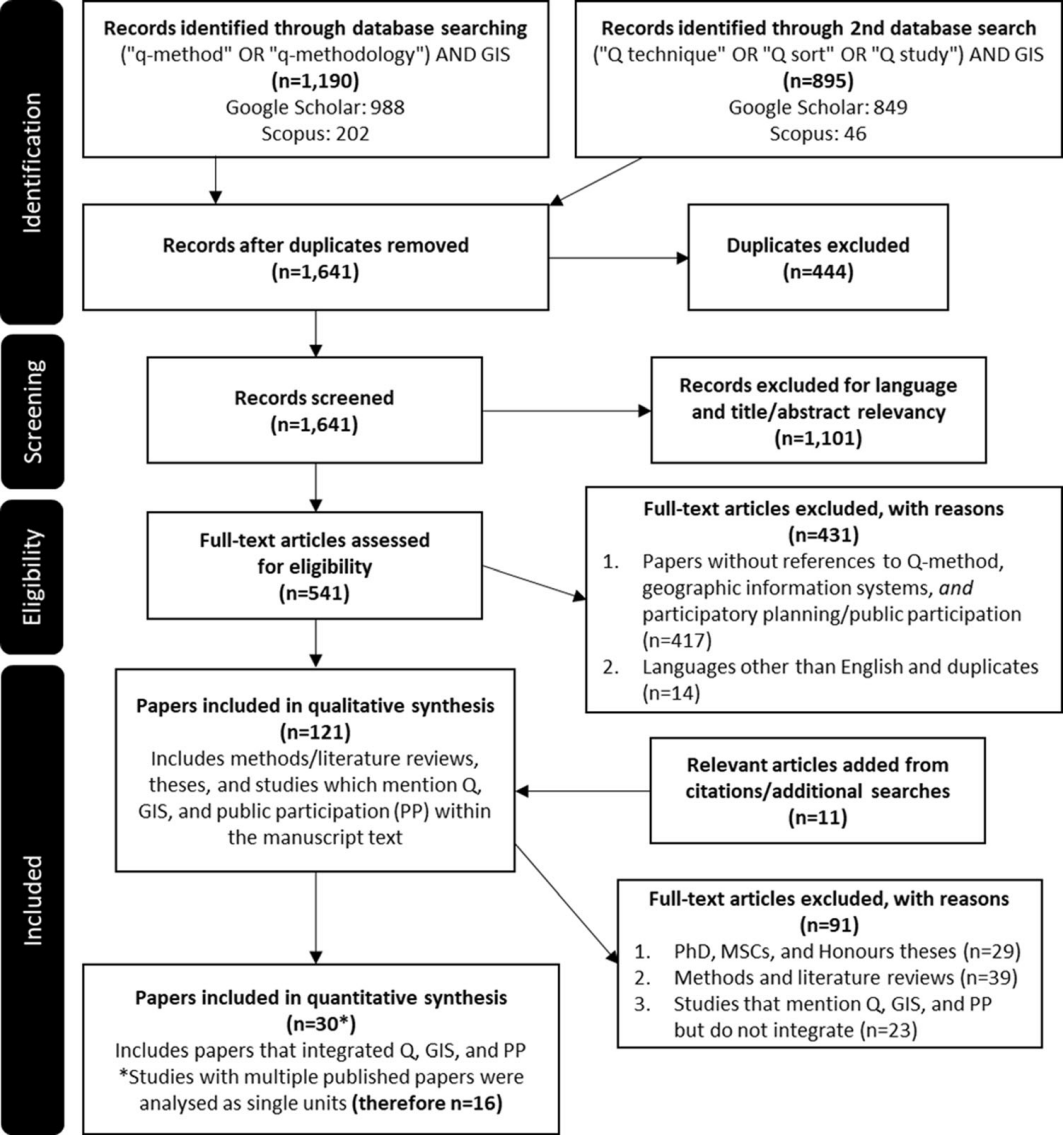
Adapted PRISMA flow chart based on Moher et al. (2009)

To answer our question—“What is the current status of research that implements the Q + PPGIS method?”, we constructed a set of concepts and associated keywords for searching the literature (Fig. 1). These are based on terminology developed through the initial steps of the SQLR process: Q-method, GIS mapping, and public participation (see Appendix S1 for a detailed account of the SQLR methodology). The specific details described in this search strategy, screening process, eligibility criteria, and the total number of studies included can be found in the Preferred Reporting Items for Systematic Reviews and Meta-Analyses (PRISMA) flow chart (Fig. 2).

An initial search was conducted in March 2020 using Google Scholar and Scopus, these search terms, (“Q method’ OR “Q methodology”) AND (PGIS or PPGIS OR “participatory mapping”), resulted in 281 papers after duplicates were removed, however only 15 papers matched the inclusion criteria of: (a) peer-reviewed papers published in English and (b) papers that reference Q and PPGIS (excluding all methods/literature reviews, theses, and other grey literature, and studies that only superficially integrated Q and PPGIS).

The initial inclusion/exclusion criteria aimed to focus exclusively on studies that combined and applied Q and PPGIS. Yet the search terms often meant papers that utilised GIS methods but did not explicitly describe methods as PPGIS or PGIS were excluded. To address this problem, another search term was applied to the databases, ("Q method" OR "Q methodology") AND GIS, that resulted in 1,190 papers between Google Scholar and Scopus in June 2020. Noticeably, what was omitted from the search terms are any direct reference to PPGIS or participatory mapping. While this second set of search terms necessitated more intense screening to determine whether or not the papers met the update inclusion/exclusion criteria, they also offered an opportunity to identify papers that were not explicit in their participatory methods but utilised some variant of public participation-based mapping.

After a thorough review of Q-method literature more generally, it became clear that an alternative set of terms have been utilised to describe the method. Therefore a third and final search term was applied, ("Q technique" OR "Q sort" OR "Q study") AND GIS, that resulted in 895 papers, with approximately half being either duplicates or in languages other than English. Only 8 of the initial 281 papers from the first set of search terms did not appear in the context of the second two, thus the exclusion of public participation/participatory from the search terms had minimal impacts on the total search results.

Given the high incidence of both theses and reviews found in the initial search, screened papers in the final search were marked for their use of GIS, Q, and public participation and its variants to determine the number of papers that used various combinations of each. The resulting 121 papers were included as part of the qualitative analysis, while only 30 papers met all of the inclusion/exclusion criteria, which is further detailed below. However, 14 of those papers were published based on data collected in a single study already included in the database necessitating analysis by studies (*n* = 16) rather than by papers.

### Eligibility criteria

After removing duplicates from the combined search results (*n* = 444), the remaining papers (*n* = 1641) were subjected to the first exclusion criteria. This entailed reading the titles and abstracts to eliminate papers that were in languages other than English and papers that were not related to the research topic (*n* = 1101). Due to the phrase “Q method”, many of the results that were excluded at this point were due to the way scholarly databases search text, where “Q” and “method” could appear adjacent but were not a reference to Q-method. Additionally, there was a high frequency of papers that referred to “Davenport’s q method”, “CODA-Q method”, and other unrelated methods that include “q method” in sequence.

The remaining papers were then subjected to the complete inclusion/exclusion criteria and keyword screening described below. The primary exclusion criteria were to ensure that papers referred to Q-method, GIS mapping, and public participation. As was often the case, the combination of search terms returned a wide range of papers that (a) only conducted Q-method or GIS mapping but included a citation that contained the other, (b) mentioned the use of either Q or GIS within the text but did not use both, or (c) were review papers/reports that included sections for both PPGIS and Q. Papers falling under the third category (c) were included as part of the qualitative analysis while papers under the second (b) were only included if the text was informative for our research question. Papers in the first (a) were excluded entirely from the review.

A subset of the excluded papers was flagged as having included notes on Q, GIS, and participation (*n* = 91). These were retained for a qualitative review for discussion and contextualisation of the SQLR results.

### Screening and study selection

To screen papers quickly and efficiently, the keywords (Fig. 1) were searched, utilising the ‘Find’ command. Once a keyword was located, the surrounding text was read for context to identify whether the study legitimately incorporated Q and PPGIS as opposed to papers that only mentioned either method.

Following inclusion/exclusion criteria application, a final list of included studies was selected. However, several papers were part of the same project or study. For example, Forrester et al. (2015) published their findings initially at a conference (Bracken et al. 2012) then later published two more papers that focused on the project’s disaster prevention and management aspects (Bracken et al. 2016; Cook et al. 2016). While the discussion section further details the multi-paper aspect of analysed studies, it is important to note that in some cases additional searches were conducted to include those multiple papers as part of the analysis. The final set of papers contained in the SQLR database identifies papers added through additional searches and studies with multiple papers (see Table 1 for the detailed database).

**Table 1:** Q + PPGIS SQLR database

ID	Title	Year	Additional search	Authors	Journal
1a	Combining participatory mapping with Q-methodology to map stakeholder perceptions of complex environmental problems	2015	No	J. Forrester, B. Cook, L. Bracken, S. Cinderby, & A. Donaldson	Applied Geography
1b	Flood risk management, an approach to managing cross-border hazards	2016	No	L. Bracken, E. Oughton, A. Donaldson, B. Cook, J. Forrester, C. Spray, S. Cinderby, D. Passmore, & N. Bissett	Natural Hazards
1c	Competing paradigms of flood management in the Scottish/English borderlands	2016	Yes	B. Cook, J. Forrester, L. Bracken, C. Spray, & E. Oughton	Disaster Prevention and Management
1d	Participatory approaches to understanding practices of flood management across borders	2012	No	L. Bracken, J. Forrester, E. Oughton, S. Cinderby, A. Donaldson, L. Anness, & D. Passmore	EGU General Assembly Conference Abstracts
2	Mapping ambivalence: Exploring the geographies of community change and rails-to-trails development using photo-based Q method and PPGIS	2008	No	T. Hawthorne, J. Krygier, & M. Kwan	Geoforum
3	Conflict mapping toward ecotourism facility foundation using spatial Q methodology	2019	No	J. Lee	Tourism Management
4	Mapping Interests by Stakeholders’ Subjectivities toward Ecotourism Resources: The Case of Seocheon-Gun, Korea	2017	No	J. Lee, S. Kim, & H. Kwon	Sustainability
5	Q-Rhetoric and Controlled Equivocation: Revising “The Scientific Study of Subjectivity” for Cross-Disciplinary Collaboration	2019	No	C. Druschke, E. Booth, & E. Lundberg	Technical Communication Quarterly
6	Why Won’t They Come? Stakeholder Perspectives on Collaborative National Forest Planning by Participation Level	2009	Yes	A. Cheng & K. Mattor	Environmental Management
7	Context matters: Agronomic field monitoring and participatory research to identify criteria of farming system sustainability in South-East Asia	2020	Yes	J. Lairez, S. Lopez-Ridaura, Damien Jourdain, G. Falconnier, P. Lienhard, B. Striffler, C. Syfongxay, & F. Affholder	Agricultural Systems
8	A multi-method approach for the integrative assessment of soil functions: Application on a coastal mountainous site of the Philippines	2020	Yes	E. Dingkuhn, A. Wezel, F. Bianchi, J. Groot, A. Wagner, H. Yap, & R. Schulte	Journal of Environmental Management
9	Ecological Landscape Planning Considering Landscape Aesthetics (Case Study: Part of Tehran-Qom Freeway)	2017	No	H. Darabi, S. Razavi, & A. Vaeziheir	Open Journal of Ecology
10	Case Puijo—Evaluation of a participatory urban forest planning process	2014	No	A. Kangas, J. Heikkilä, M. Malmivaara-Lämsä, & I. Löfström	Forest Policy and Economics
11	Mapping social-ecological systems to understand the challenges underlying wildlife management	2018	No	S. Dressel. G. Ericsson, & C. Sandström	Environmental Science & Policy
12a	Ordering Space in a Changing Climate: A Relational Analysis of Planning Practices in Bohol, Philippines	2019	No	S. Dujardin & N. Dendoncker	Planning Theory and Practice
12b	Capturing multiple social perspectives on adaptation across scales: a Q-method analysis of actors from development planning in the Philippines	2017	Yes	S. Dujardin, F. Orban-Ferauge, M.P. Cañares, & N. Dendoncker	Climate and Development
13a	The Illinois River Project and Oklahoma’s Quest for Environmental Quality	2007	No	M. Meo	Journal of Contemporary Water Research & Education
13b	Assessment and management of policy conflict in the Illinois River watershed in Oklahoma: an application of Q methodology	2007	Yes	W. Focht	International Journal of Public Administration
13c	Negotiating science and values with stakeholders in the Illinois River basin	2002	No	M. Meo, W. Focht, L. Caneday, R. Lynch, F. Moreda, B. Pettus, E. Sankowski, Z. Trachtenberg, B. Vieux, & K. Willett	Journal of the American Water Resources Association
13d	Scientists and stakeholders: Evaluating the legitimacy of the Illinois river basin management protocol	2001	No	Z. Trachtenberg	Oklahoma Politics
14	A Model for the Identification of Areas Favourable for the Development of Tourism: A Case Study of the Šumava Mts. and South Bohemia Tourist Regions (Czech Republic)	2013	No	J. Navrátil, K. Pícha, S. Martinát, J. Knotek, T. Kučera, Z. Balounová, V. White Baravalle Gilliam, R. Švec, & J. Rajchard	Moravian Geographical Reports
15a	Preferences for scenarios of land-use change in the Mackenzie/Waitaki Basin	1994	Yes	J. Fairweather & S. Swaffieldf	New Zealand Journal of Forestry
15b	Prefernces for land-use options involving forestry in the Mackenzie/Waitaki Basin	1995	No	J. Fairweather & S. Swaffieldf	New Zealand Journal of Forestry Science
15c	Planning for rural land-use change in the South Island high country	1994	No	D. Evison & S. Swaffield	New Zealand Journal of Forestry
15d	Using GIS and visualisation techniques for rural planning	1995	No	B. Hock, T. Bennison, & S. Swaffield	New Zealand Journal of Forestry
15e	Investigation of attitudes towards the effects of land use change using image editing and Q sort method	1996	No	J. Fairweather & S. Swaffieldf	Landscape and Urban Planning
16a	Indigenous voices in climate change adaptation: Addressing the challenges of diverse knowledge systems in the Barmah-Millewa	2013	Yes	D. Griggs, A. Lynch, L. Joachim, X. Zhu, C. Adler, Z. Bischoff-Mattson, P. Wang, & T. Kestin	National Climate Change Adaptation Research Facility
16b	Policy diffusion in arid Basin water management: a Q method approach in the Murray–Darling Basin, Australia	2014	Yes	A. Lynch, C. Adler, & N. Howard	Regional Environmental Change
16c	Challenges of diverse knowledge systems in landscape analysis of the Murray–Darling Basin, Australia	2017	Yes	A. Lynch, D. Griggs, L. Joachim, E. Salminen, C. Heider, T. Kestin, X. Zhu & S. Veland	Regional Environmental Change
16d	Learning from Indigenous knowledge for improved natural resource management in the Barmah-Millewa in a changing and variable climate	2014	No	D. Griggs, A. Lynch, L. Joachim, X. Zhu, C. Adler, Z. Bischoff-Mattson, P. Wang, & T. Kestin	Victorian Centre for Climate Change Adaptation Research

### Data extraction and synthesis

The remaining steps of the SQLR process involved developing a database, extracting information from publications through the screening method above, and revising categories as more papers were entered. The final SQLR database was comprised of five components: (1) where and when is Q + PPGIS utilised?; (2) what issue is being researched and why?; (3) how is the Q in Q + PPGIS implemented?; (4) How is the PPGIS in Q + PPGIS applied?; and (5) what are the key features of Q + PPGIS studies?

Once various keywords were identified in papers using the Find command, data was entered into the database about the study, using either a 1 or a 0 to signify presence/absence in addition to text descriptors on methods (such as the total number of cards included in the sort). In some cases, it was necessary to read the adjacent text in papers for keywords to confirm that the concepts were applied in the study, particularly when assessing the specifics of both methodologies. After reading additional papers, the initial set of papers were re-reviewed to complete the SQLR database.

## Results

The following results are based on the 16 studies (30 papers) that have conducted Q + PPGIS. In some cases, analysis was conducted exclusively on the 16 studies, where the results of the five multi-paper studies are compressed into a single set of results for ease in analysis. However, when applicable, the results focus on papers rather than studies, such as in the characteristics and context sub-sections. Additionally, specific papers are excluded at times from the analysis if they were deemed outliers in the context of the analysis, with explanations included as to why this occurred.

### Where and when is Q + PPGIS utilised?

Papers were published in a range of journals (only a few journals being repeated as a result of multi-paper studies). Journals spanned a range of disciplines, as determined by Scimago Journal Rankings, with (*n* = 9) falling under the *multiple discipline* category. Papers were published primarily in *environmental science* journals (*n* = 12), followed by *social sciences and geography* (*n* = 11), and *agricultural and biological sciences* (*n* = 4), and one each in *medicine* (*n* = 1), *business* (*n* = 1), and *energy* (*n* = 1).

**Fig. 3:**
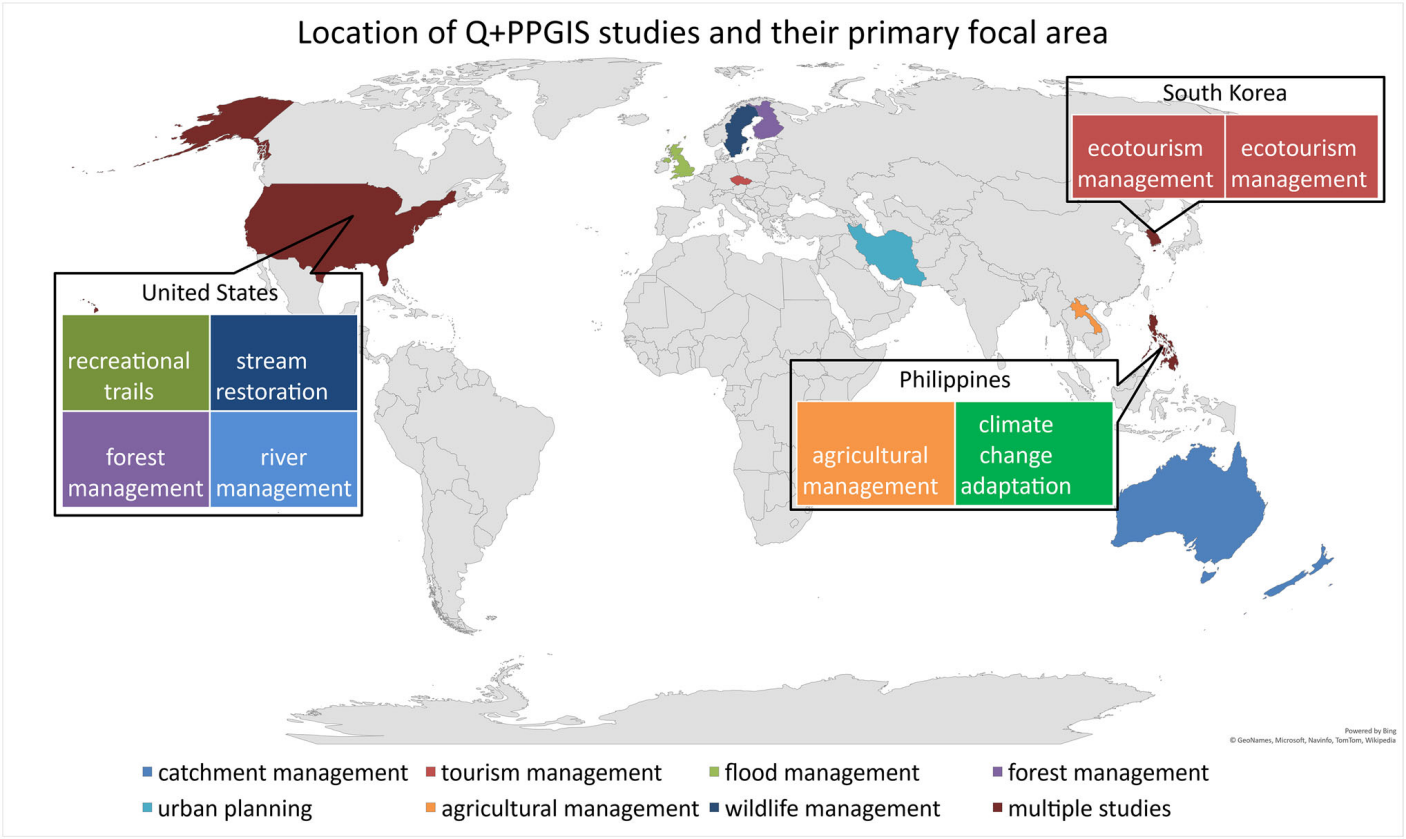
Location and primary focus of studies (*n* = 16)

Only three countries exhibited multiple Q + PPGIS studies: USA (*n* = 4), the Philippines (*n* = 2), and South Korea (*n* = 2). The remaining seven countries each only had one published (*n* = 1) Q  + PPGIS study at the time of searching. While we expected there to be relationships between the location of the study and the primary focus of the research, there appeared to be a wide range of both topics and their location (Fig. 3). Catchment management (*n* = 2) occurred only in Australia and New Zealand whereas the Korean studies (*n* = 2) were the only focus on ecotourism management. Laos and the Philippines both focused on agricultural management (*n* = 2), in addition to one Filipino study on climate change adaptation (*n* = 1). Forest management (*n* = 2) appeared in both the US and Finland with the remaining management topics (tourism, flooding, wildlife, river, recreational trails, stream restoration, urban planning) each occurring in only one country (*n* = 1 for each).

**Fig. 4:**
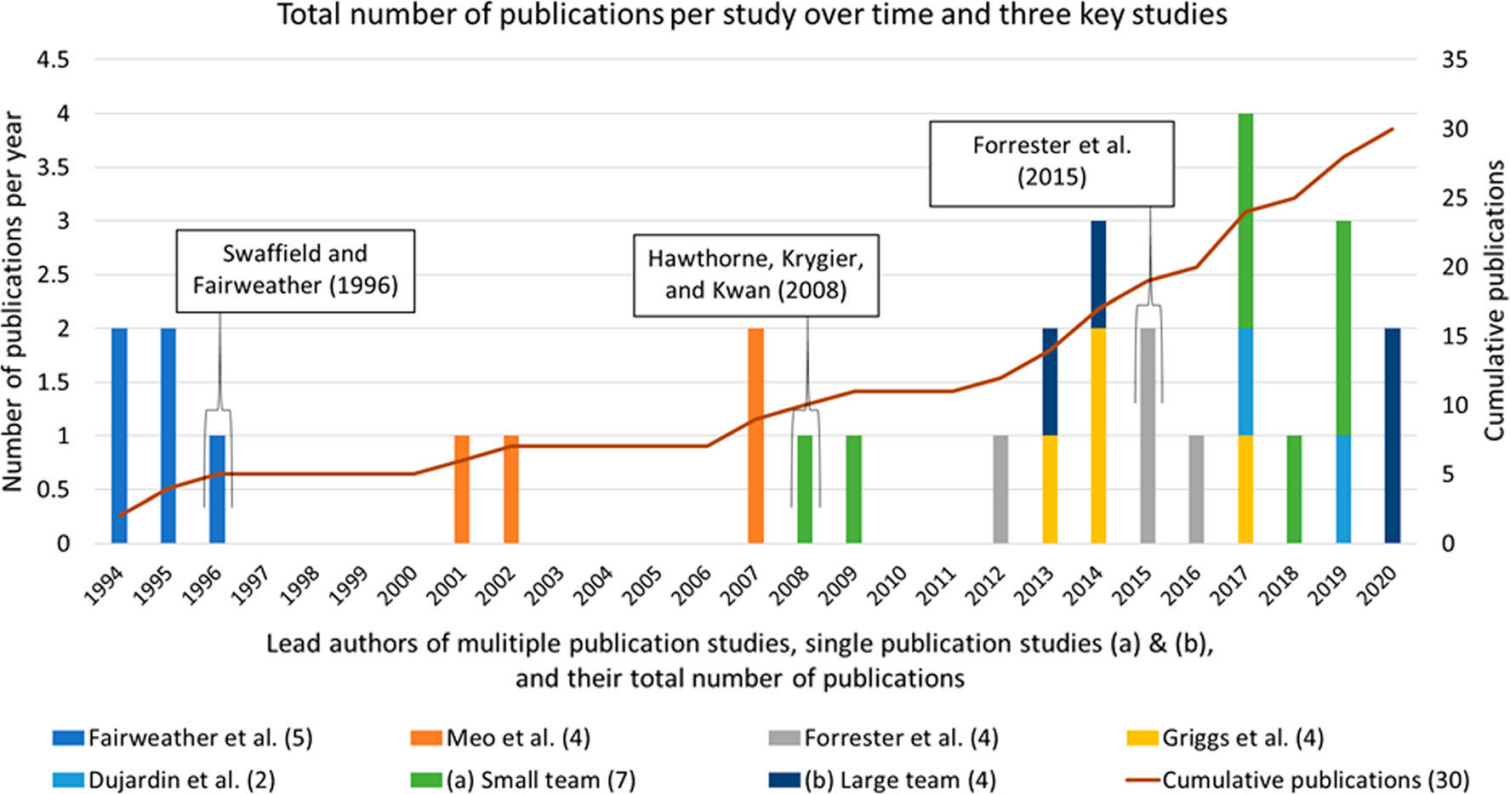
Papers per year and cumulative number of papers including studies with multiple publications, three key Q + PPGIS studies, and **a** single publication studies with small research teams and **b** single publication studies with large research teams

Most Q + PPGIS studies have been published in the last decade (*n* = 19), but there is not a clear indication that the method is increasingly being used since 2012 (Fig. 4). Five studies included multiple published papers (*n* = 19) that were clustered such that certain periods indicate noticeable jumps in the number of Q + PPGIS studies. Multiple publication studies typically consist of large research teams with four or more authors, meaning most publications included large teams (*n* = 23).

### What issue is being researched and why?

Most studies implemented the conventional methodological techniques at the core of both Q and PPGIS. With regards to the latter, only two studies did not explicitly use ‘participatory mapping’ but included some version of GIS-based approaches. Conversely, two other studies did not include any version of the term “GIS” but instead implemented ‘participatory mapping’. With the goal of stakeholder representation being central to Q + PPGIS, the two leading groups included local/community and industry (*n* = 13 and *n* = 14 respectively). Meo et al. (2002) offered the most comprehensive list of stakeholder groups: “agricultural workers (farmers), business owners, animal feeding operators (primarily poultry), nursery operators and employees, foresters, outfitters, recreationists (floaters, hikers, fishers, etc.), general recreation (secondary recreation stakeholders), local government officials, state government officials, federal government officials, Indian tribal government officials, environmentalists, journalists, and residents living near the river or lake who are not included in any of the other categories.” With most studies taking a regional/single area approach for their case study (*n* = 14), the focus on both rural (*n* = 14) and communities (*n* = 10) as well as catchments/rivers (*n* = 6), conservation areas (*n* = 6), and forests (*n* = 5) underscore the need to have a diverse set of stakeholder representation to both determine viable Q-factors and representative geospatial data (Table 2).

**Table 2:** Relationship between place context, SES concern, and spatial units

SES concern	Rural (*n* = 12) ^a^	Place context			
		Suburban (*n* = 0)^a^	Urban (*n* = 1) ^a^	Multiple (*n* = 3) ^a^	Total (*n* = 16)
Flooding	5	0	0	0	5
Land-use change	9	0	1	2	12
Soil health degradation	5	0	0	0	5
Development	8	0	1	3	12
Landslides	4	0	0	0	4
Disaster-risk	3	0	0	0	3
Run-off/LBSP	4	0	0	1	5
Human behavior	2	0	1	2	8
Spatial unit					
Catchment/basin	5	0	0	0	5
River/stream	4	0	0	1	5
Conservation area	5	0	1	0	6
Community	7	0	1	2	10
Forest/bush	1	0	1	0	4
Tourism zone/area	0	0	0	2	2

^a^The three multiple place context studies are split between rural/suburban (*n* = 2) and suburban/urban (*n* = 1), such that the results in that category would most likely apply to the suburban context, resulting in rural (*n* = 14), suburban (*n* = 3), and urban (*n* = 2)

A total of twelve different socio-ecological systems issues were addressed by the research with the most common issues being tourism (*n* = 8), agriculture (*n* = 6), climate change (*n* = 6), and water issues (*n* = 6). The future risks identified in most of the studies include development (*n* = 12), land-use change (*n* = 12), human behaviour (*n* = 7), floods (*n* = 5), soil health (*n* = 5), and land-based sources of pollution (*n* = 5), which reflects both the spatial and temporal aspects of SES (Table 2). Most study areas already had management plans in place before the study occurred *or* were conducting the study as part of a management planning process (*n* = 13). The stated goals for the management plans, the planning process, and the studies more generally included improving decision-making (*n* = 12), incorporating local knowledge (*n* = 12), evaluating the success of management plans (*n* = 10), and alleviating conflict (*n* = 6) while being part of a collaborative/cooperative policy planning process (*n* = 11). These outcomes are examples of the benefits of Q + PPGIS in responding to SES issues.

### How is the Q in Q + PPGIS implemented?

As is typical for a systematic review, we found that most studies included a definition of Q (*n* = 14) and described the method in detail (*n* = 13), with only one study not sufficiently demonstrating the application of the Q -method (Navrátil et al. 2013). Surprisingly, there were only a few references to any early Q + PPGIS publications, which demonstrates a disconnect between the method of Q + PPGIS and the larger literature. Consistent with the Q literature, statements used in the Q were generated equally (*n* = 10) from literature reviews, interviews, and from researcher landscape knowledge with only a few studies (*n* = 5) utilising pre-surveys or participatory mapping to determine the Q-concourse. While one of the earliest examples of Q + PPGIS utilized the “visual Q-method”, which is an approach that follows the same pattern as traditional Q but rather than sorting text statements participants sort images or pictures (Nazariadli et al. 2019), most studies maintained the traditional text-based statement sort (*n* = 12), with three studies employing an image-based sort. Fairweather and Swaffield (1996) were the only study in this review to use a mix of both text and images in the sorted statements, however, one text-based study noted that “visuals such as pictures/maps of the areas could be used instead of written statements, to avoid naming the areas by their zoning designation (e.g. “protection zone”) and risking influencing the participants’ judgement,” indicating a recommendation for the use of mixed statement and images (Dingkuhn et al. 2020).

To select participants, most studies reported using non-random sampling or a “semi-targeted approach” (*n* = 15), which is typical for Q, in that it ensures a representative sample of participants, known as a P-set (Griggs et al. 2014). Similarly, excluding one outlier in total participants, the average number of participants for the fifteen studies is 37 while the average number of sorted statements was 34, as is expected from Q studies that aim to a similar number of participants and statements. The choice to exclude Navrátil et al. (2013) was based on both their exceptionally high number of participants and a lack of clarity if they were participating in both the participatory mapping as well as the Q-sort. In the case of Q + PPGIS, the total number of participants for each side of the research does not need to be even, particularly given the need for PPGIS to have high levels of participants to ensure representation, whereas Q works best with smaller sample sizes.

Mimicking the stakeholder groups, many of the studies sought high diversity in their P-set by including at least three groups (*n* = 9) while only a few studies included less than two groups in their P-set (*n* = 2). The remaining studies either did not explicitly state which groups made up their P-set (*n* = 4) or only included a single group. For example, Lairez et al.'s (2020) set were spatially informed by looking at four different villages aiming for a “diversity of soil types and degree of intensification in maize cropping systems” rather than a variety of individual perspectives.

In general, most of the studies followed the traditional recommendations for conducting Q sorts. Most studies were explicit in their use of ‘forced’ sorting (*n* = 13), while the remaining three studies likely also used the ‘forced’ approach but did not describe this in their methods. More than half of the studies used a 9- or 11-point scale as part of the sort (*n* = 9) with similar levels using a combination of the Q-centric statistical analysis technique of PQMethod (*n* = 7) and the multivariate analysis method of Principal Component Analysis (*n* = 8) to analyse the results. Both of those levels are less than expected from other Q literature reviews (Dziopa and Ahern 2011). Most of the studies generated three Q-factors (*n* = 8), with many others resulting in four factors (*n* = 7), which is consistent with existing Q literature. The variance explained by the factors ranged from 52 to 88% for most of the studies (*n* = 11), with the remaining studies (*n* = 5) failing to report the variance.

### How is the PPGIS in Q + PPGIS applied?

Half of the studies described their PPGIS sessions as involving exclusively internet-based mapping programs, virtual mapping sessions, and/or the use of digital maps (*n* = 8), while fewer studies exclusively used paper maps or “low-tech” mapping techniques (*n* = 3). Two studies used a combination of paper and virtual mapping sessions, often as part of a series of sessions wherein experts and community members contribute to separate maps (Forrester et al. 2015; Griggs et al. 2014). The remaining studies (*n* = 3) did not sufficiently describe their mapping activities to allow for further analysis of their application of PPGIS. Most studies described the maps and/or the GIS process as being generated by experts with either field-specific knowledge or academic backgrounds (*n* = 10), with fewer than half of the studies depicting the maps as being citizen-generated (*n* = 4) and two studies utilising both (Table 3). Only half of all the studies (*n* = 8) demonstrated that the maps were Q-integrated (i.e., used the results of Q to either add data to the map or manipulate the map output). With the benefits of Q + PPGIS to reduce issues with each methodological approach, there appears to be a major lack of Q-integration with the mapping outputs.

**Table 3:** Associations between experts and community members in mapping and Q-sorting activities

Q-sort responsibility	Primary mapping responsibility			Total
	Community	Experts	Both	
Community	0	5	1	6
Experts	1	2	0	3
Both	3	3	1	7
Total	4	10	2	16

### What are the key features of Q + PPGIS studies?

Most studies (*n* = 14) incorporated a combination of community members and experts in either or both the Q-sort and the participatory mapping (Table 3). The two studies whose data was exclusively expert-driven were based on research questions that sought professional opinions of their problem (i.e. wildlife management and development planning) such that the multiple realities would reflect the particular stakeholder group (Dressel et al. 2018; Dujardin and Dendoncker 2019).

Most of the studies started with GIS mapping first (*n* = 7), particularly if the Q-sort was statement-based (*n* = 6), the mapping responsibility was expert-driven (*n* = 5), and if both experts and community members were involved in the Q-sort (*n* = 4). Studies that implemented Q first (*n* = 4) often included the least amount of mapping data in their results section and primarily focused on solution locations and preference change for mapping (Table 4). However, all the mapped data categories occurred at higher incidences in both the GIS-first studies and the simultaneous method order, indicating that Q often was seen as supplementary to PPGIS methods rather than central to the research study. The simultaneous methods also appeared to have an even mix of mapping responsibility, Q-sort responsibility, and an assortment of mapped data (Table 4).

**Table 4:** Total number of studies of various Q, GIS, and Q + PPGIS features in relation to the order of each method

	# of studies	Order of method		
		GIS first	Q first	Simultaneous
Q-sort type				
Statements	12	6	1	5
Photos	3	0	3	0
Both	1	1	0	0
Mapping responsibility				
Community	4	1	0	3
Experts	10	5	3	2
Both	2	1	1	0
Q-sort responsibility				
Community	6	2	3	1
Experts	3	1	1	1
Both	7	4	0	3
Mapped data^a^				
Past events	3	2	0	1
Solution location	6	3	1	2
Preference change	7	3	1	3
Landscape values	4	3	0	1
Future risk	4	4	0	0
Spatial features	5	4	0	1
Data interpretation	8	2	2	4
Total studies	16	7	4	5

^a^Totals reflect that studies had a range of 1–4 ‘mapped data’, with a mean and mode of 2.3 and 2 respectively

## Discussion

This paper aimed to investigate the application of Q + PPGIS through a systematic quantitative literature review. The findings from this systematic review found that Q + PPGIS has been applied to twelve distinct socio-ecological system challenges in multiple countries around the world by a range of disciplinary research groups indicating wide applicability of the method in answering both local and global questions. Despite the diversity of approaches, fields, and research questions observed in the Q + PPGIS literature, the method remains relatively niche with the last decade showing only a slight increase in its application. This is likely due to researchers typically not having a familiarity with the methodological approaches of both PPGIS and Q. Nevertheless, the findings show that Q + PPGIS can be readily applied to a range of SES challenges beyond what has already been identified in this literature review, especially spatially-oriented environmental planning and management processes. An identified benefit of Q + PPGIS is its ability to cross geographic and topical boundaries—suggesting the ability to scale could benefit future SES studies. With the drive towards multidisciplinary research and an increasing commitment to participatory approaches to decision-making, Q + PPGIS could enhance SES planning.

Q + PPGIS has been employed across rural, suburban, and urban settings; applied to catchment, river, forests, towns, and protected area landscapes; and has focused on issues including natural disasters, future development, land-use changes, and human behavior (Table 2). For example, Q + PPGIS has been applied with the intention to reduce conflict around flood planning and development (Fairweather and Swaffield 1996; Meo et al. 2002; Cook et al. 2016), has increased support for ecotourism facility expansion (Lee et al. 2017; Lee 2019), has improved community participation in agricultural landscape assessments (Dingkuhn et al. 2020; Lairez et al. 2020), and has enhanced understanding and support for climate change adaptation (Dujardin and Dendoncker 2019). While there remains a heavy reliance on expert opinion in all of these cases, most studies typically involve a combination of both community members and experts to drive decisions (Table 3).

While these examples demonstrate the strength of Q + PPGIS as a method for investigating SES challenges, this review has identified several concerns and potential limitations that future Q + PPGIS practitioners should address. For the method to be seen as equally relevant for both PPGIS and Q-method applications, how both methods are deployed needs to be described and demonstrated in equal measure (Table 4). This should occur in addition to explaining the integrative benefits/potentials of Q + PPGIS to a study, compared to the use of either method on its own. Better reporting of results (such as variance), better description of methods (particularly mapping activities), and improved explanation of how the approach integrates spatial, biophysical, and social data sets are essential and more reflective of muddled research rather than limitations of Q + PPGIS. Most papers we assessed contained only sparse details about their mapping procedures (*n* = 10). For example, Kangas et al. (2014) claimed they conducted “participatory mapping” as part of their study but did not detail the methodology sufficiently for evaluation (and replication). Similarly, there is a need for better consistency in reporting the details of results arising from the use of Q + PPGIS, exemplified by the five studies we have identified that did not report the explained variance.

The implementation of Q in the studies we assessed also has drawbacks, consistent with the existing literature on Q-method (Eden et al. 2005). For example, Hawthorne et al. (2008) indicated that the picture sorts used for their data collection often presented vague meanings that led to multiple interpretations from the researchers. This could be overcome by either implementing Fairweather and Swaffield’s (1996) combined text and image cards or by acknowledging the Q-sorts as more of a rhetorical method with existing limitations (Druschke et al. 2019). Kangas et al. (2014) found that the use of Q-method was seen both as complicated enough to reduce both participation and enjoyment for willing participants, although other Q-studies have found the process to have features of gamification making it an entertaining experience (Lutfallah and Buchanan 2019; Nazariadli et al. 2019), indicating this may be more of a limitation for particular studies or participants rather than of Q + PPGIS as a whole.

Furthermore, Q does not provide results concerning proportions of the population, unlike standard “R method” correlations, and tends to be a time-consuming method that can limit its value for spatial planning and SES issues (Fairweather and Swaffield 1996; Eden et al. 2005; Danielson 2009). However, the targeted use of public participation techniques can elicit this population data without sacrificing the value-add of Q (Griggs et al. 2014). Additionally, while the Q process is likely to remain time-heavy, effective development of a Q-concourse allows for study replication across spatial and temporal scales, which can enhance existing spatial planning approaches (Fairweather and Swaffield 1996; Lairez et al. 2020). Consequently, our findings indicate that Q + PPGIS can be useful for a range of topics, in various locations, across different scales and in different settings (urban, suburban, rural, flowing, growing, etc.), demonstrating a potential to supersede the limitations of either method used autonomously.

Importantly, there remains a gap in the literature for a cohesive definition and description of Q + PPGIS, which this review has sought to resolve. Drawing upon existing studies, we have defined Q + PPGIS as a *mixed-method approach that employs both Q and GIS as part of public participation planning processes*. The main benefit of Q + PPGIS is its ability to geographically situate and represent the multiple realities of individuals within a community to reduce conflict around SES challenges. While it is unlikely that a unified step-by-step description of the method can be determined due to the diversity of steps, phases, and responsibilities of each contributing method, this review has identified a range of methodological approaches that future Q + PPGIS practitioners can consider descriptive of Q + PPGIS. Moreover, by bringing these papers together, we have identified gaps in the literature regarding the application of Q + PPGIS. We hope this will drive momentum for applying the method more widely in response to SES challenges, building on the strengths and redressing the limitations discussed below.

## Limitations and future research

Due to the nature of the SQLR method, there are some limitations in the results that give rise to future research opportunities. First, we identified an additional 23 papers that included a mention of both Q and PPGIS as part of their methods or utilised both Q and GIS but did not sufficiently meet the definition of Q + PPGIS presented in this review. For example, Fry et al. (2017) used GIS to develop maps of shale gas wells and took nine aerial photographs to use as part of their Q-sort to identify perspectives on the arrangement of production sites. While their research appeared to be Q + PPGIS, the GIS mapping did not necessarily fit any participatory methods as it relied exclusively on spatial scientists. A further investigation into these non-Q + PPGIS papers would help to delineate the method more effectively.

Second, even within the papers included in our systematic review, five of the studies demonstrated features that could potentially disqualify them as Q + PPGIS studies, depending on the methodological definition employed. This includes Darabi et al. (2017), who purport to conduct a Q-sort, but there is not clear evidence that the method they employed was a traditional Q study; Kangas et al. (2014) who used their sort to analyse a participatory mapping-based method rather than integrating Q as part of their planning process; Dujardin et al. (2018) who employed a process where GIS was used to generate hazard maps through public participation with unclear details on their mapping process; and Navrátil et al. (2013) who used Q to sort landscape features and future scenarios then mapped the results based on the features themselves, as opposed to conducting participatory mapping activities. Future research needs to better elucidate Q + PPGIS approaches employed, to remove confusion about the use of the method.

Third, we also identified several PhD/Masters theses (*n* = 29) and method reviews (*n* = 39) directly related to Q + PPGIS that were excluded from the analysis but require discussion. This further demonstrates the increasing interest in the method, especially for PhDs who can easily publish multiple papers from a Q + PPGIS study, such as Vaas et al. (2019). PhDs generally have the benefit of involving multiple researchers focused on a particular issue taking a few years, which often supports the structure of the mixed-method. With regards to the method reviews, these papers tended to include both Q and PPGIS in lists about potential participatory methods. For example, Maxwell (2018) detailed that Q-method was effective in representing "knowledge, values, worldviews, & beliefs" while PPGIS uncovers a "sense of place and identity." Similar themes were mentioned in Nijnik and Miller's (2017) review of participatory mixed-methods in planning for ecosystem services where Q identifies "patterns in stakeholder perspectives" and PPGIS analyses the “public perception of landscape features.”

Fourth, since this review excluded non-English papers (*n* = 14), including papers in Mandarin, German, Farsi, Italian, and Korean from similar disciplines comprising sustainable tourism, urban planning, renewable energy, sustainable development, cultural ecosystem services, and spatial planning. In addition to the tendency towards discipline-specific terminology, it is possible that the use of Q + PPGIS may be more widespread than our findings indicate. Overall, the papers, reviews, and theses not analysed in this review indicate an appetite for Q + PPGIS and the potential for its further publication in a wide range of disciplines working on SES issues.

In considering future research, it would be helpful to fully investigate the seminal papers utilising Q + PPGIS to better track the development of the method over time, specifically Pitt and Zube (1979) as well as Swaffield and Fairweather (1996). As detailed in this review, several studies have specified the method much better than others. By investigating those papers, Q + PPGIS can be better placed in relation to both PPGIS and Q-method research. Another area of future research should be the specific approaches (i.e., order of methods and integration extent) of Q + PPGIS. While this paper was able to shed light on the similarities and differences between the literature (Table 4), it was beyond the scope of this review to speculate on these approaches without providing more context on each individual paper. Future research should further explore the grey literature to determine how non-peer-reviewed studies are employing Q + PPGIS. Lastly, given the relevance of SES theory within the Q + PPGIS framework, there is a need for similar systematic reviews on the intersection of Q and SES and PPGIS and SES as well as their intersections within other theoretical approaches that have not yet been subject to systematic literature reviews.

## Conclusion

Mixed-methods approaches that are both participatory (responding to the values and perceptions of communities) and integrative (merging spatial, social, and environmental datasets) are essential to understanding complex SES challenges. Two distinct methods are increasingly employed based on their abilities to cross the quantitative and qualitative divide: public participation GIS and Q-method (Craig et al. 2002; Eden et al. 2005). PPGIS is a powerful tool that incorporates community responses with key geospatial data to provide spatially-grounded policy recommendations and is best used as part of a suite of methods (Nijnik and Miller 2017; Maxwell 2018; Ungar et al. 2020). Q-method is particularly appropriate in supporting PPGIS, because it is useful for revealing stakeholder perceptions and values of topics, which can result in policy appraisal, land-use management alternatives, critical reflection, and conflict resolution (Mukherjee et al. 2018). By combining Q and its ability to reveal individual agendas with PPGIS and its rigour in exposing community, political, and social issues, the resulting mixed-method of Q + PPGIS is a potentially effective approach that is currently underutilised in the SES literature and more broadly (Harris and Weiner 2002; Danielson et al. 2012).

This paper has defined and described Q + PPGIS through a systematic quantitative literature review. In doing so, the use of the method was mapped over time, the application of the method assessed, and the key similarities and differences between studies were determined, presenting a comprehensive overview of the applications, strengths, and limitations of the method. Both Q and PPGIS are widely utilized tools that have unique benefits and challenges in their pursuit in exploring complex SES challenges. When applied as a mixed-method, they can expose the association between discourses and place-based knowledge, which is essential for SES planning, management, and decision-making. By detailing the similarities and differences between Q + PPGIS methods in the current literature, we have shown that Q + PPGIS often occurs as part of large research groups in the context of multidisciplinary or transdisciplinary research and that the method can be applied to a wide range of topics, disciplines, and system issues. However, the present lack of specific disciplinary focus, as well as the diversity in methodological approach, could be limiting the broader application of Q + PPGIS. We believe the method has considerable potential and its application is worthy of greater attention from scholars and practitioners alike. By recognising the multiple realities surrounding socio-ecological system challenges, Q + PPGIS allows communities to both facilitate more rigorous theories of change and ensure a deeper understanding of the complex coupled human-natural systems both in the present and into the future.

## Supplementary Information

Below is the link to the electronic supplementary material.

Supplementary file1 (PDF 1085 kb)
