# Supplementary material for: The benefits of Q + PPGIS for coupled human-natural systems research: A systematic review
Source: Ambio. 2022 Mar 7;51(8):1819–36. doi: 10.1007/s13280-022-01709-z (PMC9200925; doi:10.1007/s13280-022-01709-z)
Supplement: Supplementary file 1 — Supplementary file1 (PDF 1085 kb) [file 13280_2022_1709_MOESM1_ESM.pdf]

Ambio

Supplementary Information

This supplementary information has not been peer reviewed

Title: **The benefits of Q+PPGIS for coupled human-natural systems research: a systematic review**

Appendix S1: Detailed systematic quantitative literature review methodology

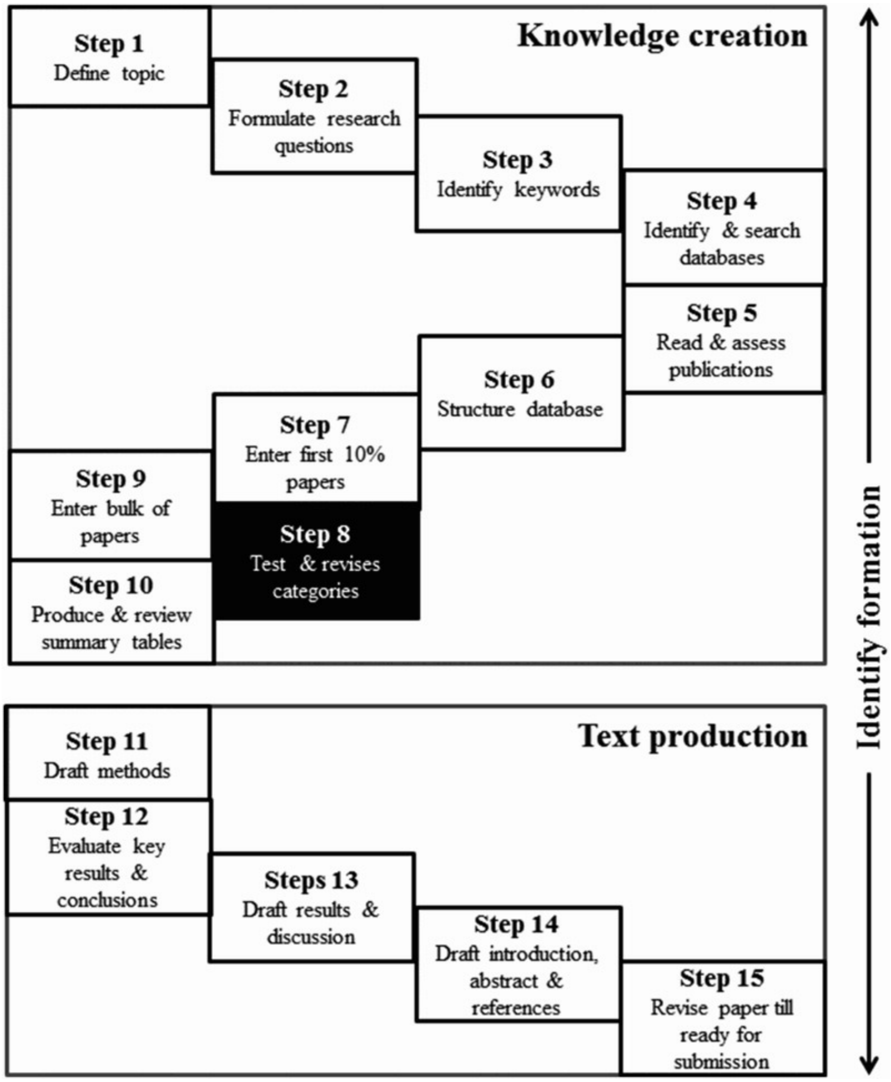

Figure S1: Steps involved in undertaking a systematic quantitative literature review in relation to the process of knowledge creation, text production and identify formation (from Pickering et al., 2015)

Step 1

The initial step of an SQLR process is to define the topic for the research. Based on an initial inventory, the two leading papers that have implemented Q+PPGIS are Forrester et al. (2015) and Hawthorne et al. (2008). The former was responsible for coining the term “Q+PPGIS” and described the benefit in the method to address the challenge that “maps alone lack the ready inclusion of social values and beliefs that are necessary for understanding socio-environmental, and therefore socio-political, elements” (Forrester et al. 2015). The latter was cited as a leading influence in combining the two methods by providing rationale that “Q-method can be used to identify [commonalities of opinions], which can then be mapped in GIS and used to strengthen existing spatial data for use in planning discussions” (Hawthorne, Krygier, and Kwan 2008). While these key

texts allude to similar methodological approaches (Fairweather and Swaffield 1996; Pitt and Zube 1979), it became clear that the full extent of Q+PPGIS research had not yet been documented. Consequently, the primary topic of inquiry in this review is around studies that have implemented Q+PPGIS research.

Step 2

Continuing based on the selected topic, step two of an SQLR requires the identification of research questions that will be addressed through a systematic review (Section 1.1.). The formulation of the research questions for this research involved an iterative process throughout the data collection stage of the literature review. As more papers were assessed for eligibility and reviewed for inclusion in the primary database, themes in the data started to emerge that shed light on the direction the review needed to follow. Specifically, RQ4 was generated in response to the apparent lack of widespread implementation of Q+PPGIS and thus aims to provide recommendations within the discussion section of this paper to further utilize the methodology.

Step 3

The third step of the SQLR process, prior to searching databases and selecting studies for inclusion, necessitates the identification of keywords. For this study, the three central components of a Q+PPGIS study are Q-method, GIS mapping, and public participation, as demonstrated in the Q+PPGIS conceptual framework (Figure 1). However, as is the case for searching literature as part of a review process, relying exclusively on three keywords can limit the accuracy of included papers. Therefore, the central components are expanded to allow both for ease in screening as well as for more precisely defining concepts.

Q-methodology – Typically, studies that has implemented the Q-method will explicitly detail the method either in the abstract or in the methods section. It was determined after reviewing Q literature more generally that in some cases synonyms for the term Q-method were used, including Q-sort, Q-technique, or Q-study. Less frequently, rather than repeating the method terminology, papers focus on the process or output of Q; the former includes the sorting and card(s) making up the fourth step of a Q process while the latter refers to discourse and factor(s) that represent the typical output terminology post-analysis.

GIS mapping – The use of geographic information systems in research is widespread, yet it takes many forms when described in the literature. While some studies mention the use of ArcGIS (or similar software), many describe the use of GIS through related terms like map(s), spatial, and geographic. Searching for mapping or sketch mapping was problematic because of the tendency in Q

literature to describe the process as [discourse, stakeholder, causal, or cognitive] mapping. Additionally, while the initial search focused on PPGIS and PGIS, the integration of GIS and Q does not explicitly necessitate the use of either.

Public participation – Using both Q-method and GIS as part of a study does not necessarily mean Q+PPGIS was implemented. PPGIS necessitates public participation during the mapping/GIS method as opposed to researcher conducted GIS mapping. As such, it was important to search for participatory and participation in addition to Q and GIS. In some cases, a focus on the stakeholder or community and their role in providing an assessment or their perception of the geospatial data was satisfactory in confirming the presence of public participation.

The following details steps four through nine of the SQLR process (Pickering and Byrne 2014). The adapted PRISMA diagram (Figure 2) demonstrates the identification/search strategy, screening process, eligibility criteria, and total number of studies included as part of the meta-analysis. While systematic reviews are particularly useful for analysing large data sets, this paper offers a comprehensive review of Q+PPGIS studies such that demonstrates the underutilization of the method while presenting the numerous benefits of combining Q and PPGIS.

Step 4

When identifying and examining databases an initial search was conducted in March 2020 using Google Scholar and Scopus with the search terms:

(“Q method’ OR “Q methodology”) AND (PGIS or PPGIS OR “participatory mapping”)

The search resulted in approximately 281 papers after duplicates were removed, however only 15 papers matched the inclusion criteria of i) papers in English and ii) papers that reference Q and PPGIS, while excluding all methods/literature reviews, theses, and other grey literature, and studies that did not sufficiently integrate Q and PPGIS. For example, Hugé et al. (2016) mention that “other methods (such as… participatory mapping…) would be needed,” yet did not incorporate any PPGIS methods as part of their study. Alternatively, it became clear that the use of both “Q-method” and “Participatory mapping” were being regularly mentioned in literature reviews, such as Nkoana, Verbruggen, and Hugé's (2018) review of community climate change adaptation tools.

The initial inclusion/exclusion criteria aimed to focus exclusively on studies that combined and applied Q and PPGIS, yet the initial search terms often meant papers that utilized GIS methods but not described explicitly as PPGIS or PGIS were excluded entirely. In order to address this gap in the review approach, another search term was applied to the databases.

("Q method" OR "Q methodology") AND GIS

This second search query resulted in 1,190 papers between Google Scholar and Scopus in June 2020. Noticeably, what is excluded from the search terms are any direct reference to PPGIS or participatory mapping. While this second search terms necessitated more intense screening in order to determine whether or not the papers met the update inclusion/exclusion criteria, they also offered an opportunity to identify papers that were not explicit in their participatory methods but utilized some variant of human ecology mapping. However, after a thorough review of Q-method literature more generally, it became clear that an alternative set of terms were utilized to describe the Q method, thusly a third and final search term was applied:

("Q technique" OR "Q sort" OR "Q study") AND GIS

This final search resulted in 895 papers, however, approximately half of them were either duplicates or in languages other than English. Only 8 of the initial 281 papers from the first set of search terms did not appear in the context of the second two, thus the exclusion of “PP” from the search terms had minimal impacts on the total search results. Given the high incidence of both theses and reviews found in the initial search, screened papers in the final search were marked for their use of GIS, Q, and public participation (PP) and its variants in order to determine the number of papers that used various combinations of each. The resulting 121 papers were included as part of the qualitative analysis, while only 16 studies met the entirety of the inclusion/exclusion criteria.

After removing duplicates from the combined search results (n=444), the remaining papers (n=1,641) were subjected to the first exclusion criteria, which involved reading the titles and abstracts to eliminate papers that were in languages other than English and papers that were clearly not related to the research topic (n=1,101). Due to the phrase “Q method”, many of the results that were excluded at this point were due to the way databases search text where “Q” and “method” could appear adjacent but were not a reference to Q-method. Additionally, there was a high frequency of papers that referred to “Davenport’s q method”, “CODA-Q method”, and other unrelated methods that include “q method” in sequence.

The remaining papers were then subjected to the complete inclusion/exclusion criteria and keyword screening described below. The primary exclusion criteria were to ensure that papers made references to Q-methodology, GIS mapping, and public participation. As was often the case, the combination of search terms returned a wide range of papers that (a) only conducted Q-method or GIS mapping but included a citation which contained the other, (b) mentioned the use of either Q or GIS within the text but did not use both, or (c) were review papers/reports that included sections for

both PPGIS and Q. Papers falling under the third category (c) were included as part of the qualitative analysis while papers under the second (b) were only included if the text was informative to this study. Papers in the first (a) were excluded entirely from the review.

A subset of the excluded papers were flagged as having included notes on Q, GIS, and participation (n=91). Thus these were retained for a qualitative review for discussion and contextualization of the SQLR results.

Step 5

In order to screen papers quickly and efficiently, the keywords and associated words identified in Figure 1 were combed in papers utilizing the 'Find' command. Once a keyword was located, the surrounding text was read for context in order to ensure that the study legitimately incorporated Q and PPGIS as opposed to papers that only mention either method.

Following inclusion/exclusion criteria application, a final list of included studies was settled upon. However, there were a number of papers that were part of the same project or study. For example, Forrester et al. (2015) published their findings initially at a conference (Bracken et al. 2012) then procced to publish two more papers that focused on the project's disaster prevention and managmgnet aspects (Bracken et al. 2016; Cook et al. 2016). While the discussion section further details the multi-paper aspect of analysed studies, it is important to note that in some cases additional searches were conducted in order to include those multiple papers as part of the analysis. The final set of papers included in the database can be found in Appendix A, which includes papers added through additional searches and studies with multiple papers.

Step 6 onward

The remaining steps of the SQLR process involve developing a database, extracting the data through the screening method above, and revising categories as more papers are entered. A database was generated which focused on five sections: (1) characteristics of the study, (2) research context, (3) application of Q, (4) application of PPGIS, and (5) the method integration/outputs.

Once various keywords were identified in papers through the Find command, either a 1 or a 0 was entered into the database to signify presence/absence in addition to specifics on methods (such as the total number of cards included in the sort). In some cases, it was necessary to read adjacent text for certain keywords to confirm that the concepts were applied in the study, particularly when assessing the specifics on both methodologies. After reading additional papers, the initial set of papers were re-reviewed in order to fill in any blanks in the database.

For the results and analysis, see the main text.
